# Childhood Immunization Gaps in New York State After COVID-19: A Focus on Regional Disparities

**DOI:** 10.7759/cureus.91850

**Published:** 2025-09-08

**Authors:** Katrene Rustemi, Varun Soti

**Affiliations:** 1 Pediatrics, Lake Erie College of Osteopathic Medicine, Elmira, USA; 2 Pharmacology and Therapeutics, Lake Erie College of Osteopathic Medicine, Elmira, USA

**Keywords:** childhood vaccination, covid-19 pandemic, immunization coverage, measles mumps rubella (mmr), new york city, new york state, vaccine hesitancy

## Abstract

Introduction

Vaccination is one of the most effective public health measures for preventing infectious diseases. However, the pandemic of the coronavirus disease 2019 (COVID-19) disrupted routine immunization services worldwide, raising concerns about reduced childhood vaccination coverage. New York State (NYS), particularly New York City (NYC), experienced significant pandemic-related challenges that may have impacted pediatric immunization rates.

Materials and methods

This study analyzed childhood vaccination coverage in NYS using the Centers for Disease Control and Prevention’s data. It compared the vaccination coverage during the pre-pandemic period (2018-2019) to the post-pandemic period (2020-2021). Vaccination rates for nine recommended vaccines by 24 months of age were examined across three geographic categories: NYS overall, NYC, and the rest of NYS (excluding NYC). Statistical analyses included one-way analysis of variance with Tukey’s post hoc test, with significance at a probability value (or p) less than 0.05.

Results

In NYS overall, vaccination rates declined slightly from 80.29% pre-pandemic to 78.97% post-pandemic (p = 0.6008). The rest of NYS (excluding NYC) showed a similarly modest decline (80.19% to 79.85%, p = 0.9985). In contrast, NYC experienced a significant reduction from 80.41% to 77.82% (p = 0.0288). Measles, mumps, and rubella (MMR) vaccination rates declined statewide, with coverage falling from 93.30% to 89.60% (p = 0.0459). In NYC, the decline was particularly pronounced, with MMR coverage dropping from 93.30% to 86.70% (p = 0.0039). Post-pandemic comparisons revealed significant disparities between NYC and the rest of NYS (p = 0.0128).

Discussion

The findings indicate that the pandemic disproportionately affected vaccination coverage in NYC, likely due to prolonged lockdowns, healthcare access barriers, and heightened vaccine hesitancy. While overall declines were modest, reductions in MMR coverage are particularly concerning given the resurgence of measles. The results underscore the importance of regionally tailored recovery strategies, including catch-up vaccination campaigns, improved healthcare delivery systems, and interventions to counteract misinformation.

Conclusions

The COVID-19 pandemic caused a measurable decline in childhood vaccination rates in NYS, with NYC experiencing the most significant reductions. Restoring vaccine coverage requires targeted interventions to strengthen healthcare resilience, rebuild public trust, and prevent future outbreaks of vaccine-preventable diseases.

## Introduction

Children inevitably come into contact with various germs in their everyday environments. Vaccination is critical in safeguarding children’s health because such exposures are unavoidable [1]. Since their introduction, vaccines, particularly those administered in early childhood, have been among public health’s most effective tools for preventing infectious diseases [2]. Because infants are born with immature immune systems, vaccinations provide a safe and reliable way to train the body’s immune defenses to recognize and combat serious pathogens [3].

In the United States (US), standard childhood immunizations include vaccines against diphtheria, tetanus, and pertussis (DTaP); polio; measles, mumps, and rubella (MMR); *Haemophilus influenzae* type B (Hib); hepatitis B; varicella (chickenpox); and pneumococcal disease. The effectiveness of these vaccines is apparent: from the 20th century to 2024, the rates of illnesses prevented by vaccines, including measles, mumps, diphtheria, rubella, and Hib, have declined by more than 99% [4,5].

Despite this success, a recent resurgence of measles in the US has raised serious public health concerns [6,7]. According to the Centers for Disease Control and Prevention (CDC), as of May 2025, there have been 12 measles outbreaks nationwide, resulting in 935 confirmed cases. These outbreaks have occurred across various states, including New York. In contrast, in all of 2024, there were only 16 outbreaks. Alarmingly, 96% of the 2025 cases occurred in unvaccinated children or those with unknown vaccination status. To date, three measles-related deaths have been confirmed [8].

The continued decline in national childhood vaccination coverage heightens the concern [9,10]. The CDC reports that vaccination rates among kindergarten-aged children have dropped below 93% for all recommended vaccines, down from 95% during the 2019-2020 school year in the US. Simultaneously, immunization exemption rates have risen to 3.3%, with 14 jurisdictions reporting rates exceeding 5% [11]. These trends increase the risk of future outbreaks of vaccine-preventable diseases, highlighting the urgent need for coordinated public health responses [12].

One significant factor contributing to the decline in vaccination rates is the pandemic of the coronavirus disease 2019 (COVID-19) [9,13]. Beginning in early 2020, the pandemic caused widespread disruptions to routine childhood immunization services across the US. The federally funded Vaccines for Children (VFC) program experienced a sharp decline in vaccine orders between January and April 2020, notably after the US government declared a national emergency in March. This trend affected all non-influenza vaccines recommended by the Advisory Committee on Immunization Practices, including those for measles. Vaccine Safety Datalink data revealed a substantial drop in vaccine administration, especially among children over 24 months of age [13].

Contributors to this decline included clinic closures, altered healthcare workflows, and parental concerns about COVID-19 exposure during in-person visits. While there were efforts to prioritize vaccinations for younger children (≤24 months), the overall reduction raised significant concerns about growing vulnerability to vaccine-preventable diseases. Another factor contributing to these declines is the misinformation surrounding vaccinations that emerged during the rollout of COVID-19 vaccines throughout the pandemic. This misinformation has led to increased mistrust in the healthcare system. False claims, such as the idea that COVID-19 vaccines cause monkeypox or cancer, and assertions that the US Food and Drug Administration is intentionally withholding more effective vaccines from the public spread rapidly through social media [14].

Furthermore, a poll from the Kaiser Family Foundation showed that a significant percentage of adults and parents reported encountering false claims regarding vaccinations, including the widely disseminated misinformation that the MMR vaccine causes autism and that measles vaccines are more dangerous than the disease itself. This exposure to misinformation has led to increased uncertainty, as many adults expressed doubts about the safety and necessity of vaccines. While a few believed these false claims to be valid, a substantial number were uncertain about their veracity. Notably, parents who leaned toward believing these misconceptions were more likely to delay or skip recommended vaccines for their children, highlighting the profound impact of misinformation on vaccination decisions [15-18].

Globally, the disruption was even more severe. According to the World Health Organization and United Nations International Children’s Emergency Fund, 23 million children worldwide missed basic childhood vaccinations in 2020, the highest number since 2009 and 3.7 million more than in 2019. This decline stemmed from strained healthcare systems, lockdowns, and limited access to routine health services [19].

Given that New York City (NYC) was a major epicenter of the COVID-19 pandemic in the US, this study examined how the pandemic affected childhood vaccination rates in this region. The study aimed to assess the impact of the pandemic on childhood vaccination coverage in New York State (NYS) by exploring the following key objectives: (1) It sought to understand the overall effect of the COVID-19 pandemic on childhood vaccination rates in NYS and specifically in NYC. (2) The study assessed the impact on childhood vaccination rates in the rest of NYS, excluding NYC. (3) The study evaluated how the pandemic impacted vaccination rates in NYC compared to the overall NYS and the rest of NYS. (4) It compared the impact of the pandemic on childhood vaccination rates across NYS with those in the rest of NYS, excluding NYC.

The study findings could be valuable in guiding targeted interventions and informing policy decisions to enhance vaccination coverage. By identifying specific regions within NYS that experienced significant declines during the COVID-19 pandemic, public health authorities and clinicians can allocate resources more effectively, launch catch-up immunization campaigns, and address barriers to access. Moreover, a deeper understanding of these patterns may assist clinicians in identifying and engaging populations at higher risk of vaccine-preventable diseases, fostering proactive patient education and preventive care in both clinical and community environments.

## Materials and methods

Data collection and extraction

Vaccination coverage data for this study were obtained from the CDC ChildVaxView interactive data tables [20] on March 17, 2025. To assess the impact of the COVID-19 pandemic on childhood immunization, birth cohorts from 2018 and 2019 were selected to represent the pre-pandemic period, while birth cohorts from 2020 and 2021 represented the post-pandemic period. To ensure consistency in vaccine uptake across comparable age ranges, the dataset was limited to coverage by 24 months [21].

Regional vaccination data were extracted for three distinct geographic categories within NYS, the rest of NYS (which excluded NYC), and the overall NYS. The analysis focused on a set of routine childhood vaccinations that are recommended for children by the age of 24 months. These vaccinations include the MMR vaccine, DTaP vaccine, rotavirus vaccine, hepatitis A vaccine, hepatitis B vaccine, Hib vaccine, pneumococcal conjugate vaccine, and inactivated polio vaccine [21].

Statistical analysis

For each region, the averages of the vaccination rates for the pre-pandemic and post-pandemic periods were calculated. Descriptive statistical analyses and data visualizations were conducted using GraphPad Prism (Dotmatics, Boston, MA, US) for the overall and MMR vaccination rates. A one-way analysis of variance was performed to assess whether statistically significant differences existed in average vaccine coverage between geographic groups and periods [22]. Subsequently, Tukey’s multiple comparisons test was used for post hoc analysis to identify specific group differences. A significance level of probability value (or p) less than 0.05 was used to determine statistical significance throughout the analysis [23].

## Results

This study analyzed childhood vaccination rates for nine recommended childhood vaccines. It focused on comparing the periods before the COVID-19 pandemic (2018-2019) and after the pandemic began (2020-2021). The analysis was conducted across three distinct regions: NYS overall, NYC, and the rest of NYS (excluding NYC). The sample size for the number of children's records for nine routine childhood vaccinations in NYS overall was 1,370 pre-pandemic and 1,026 post-pandemic. For the rest of NYS, excluding NYC, it was 507 before the pandemic and 466 afterward. For NYC, the pre-pandemic sample size was 863, while post-pandemic, it was 560.

Childhood vaccination rates for nine recommended vaccines in NYS pre- and post-pandemic

In NYS overall, the average childhood vaccination rates for the nine vaccines declined from 80.29% before the pandemic to 78.97% following the pandemic. However, this change was not statistically significant (p = 0.6008), resulting in a mean difference of 1.33%. In the regions of NYS outside of NYC, the vaccination rate experienced a slight decrease from 80.19% pre-pandemic to 79.85% post-pandemic. Again, this difference was not statistically significant (p = 0.9985), yielding a mean difference of 0.34%. In contrast, within NYC, the average vaccination rate declined from 80.41% before the pandemic to 77.82% thereafter. This change was statistically significant (p = 0.0288), reflecting a mean difference of 2.59%, which indicates a marked reduction in childhood vaccination rates in NYC. Please refer to Table 1 for further details.

**Table 1:** Pre- and post-pandemic childhood vaccination rates for nine vaccines in NYS, NYC, and the rest of NYS (excluding NYC). This table displays average childhood vaccination rates for nine routine immunizations before (2018-2019) and after (2020-2021) the onset of the COVID-19 pandemic. Data are reported separately for NYS overall, NYC, and the rest of NYS (excluding NYC). *The p-value was considered statistically significant if it was less than 0.05. Sample sizes: pre-pandemic NYS overall = 1,370; post-pandemic NYS overall = 1,026; pre-pandemic rest of NYS = 507; post-pandemic rest of NYS = 466; pre-pandemic NYC = 863; post-pandemic NYC = 560. =: equal; %: percent; CI: confidence interval; COVID-19: coronavirus disease 2019; NYC: New York City; NYS: New York State; p: probability.

Region	Pre-pandemic (%)	Post-pandemic (%)	Mean difference (%)	95% CI	p-value
NYS overall	80.29 (71.78-88.81)	78.97 (69.91-88.03)	1.33	-1.09 to 3.75	0.6008
Rest of NYS	80.19 (71.13-89.25)	79.85 (70.56-89.14)	0.34	-2.08 to 2.76	0.9985
NYC	80.41 (72.46-88.36)	77.82 (68.91-86.73)	2.59	0.17 to 5.01	0.0288*

Pre-pandemic regional differences in vaccination rates for all nine childhood vaccines revealed no statistically significant differences between NYC, the rest of NYS, and the overall NYS. Similarly, post-pandemic vaccination rates also showed no significant discrepancies across these regions (see Table 2).

**Table 2:** Regional childhood vaccination rates for nine vaccines in NYS with pre- and post-pandemic comparisons. This table presents comparative analyses of average childhood vaccination rates for nine routine vaccines between regions (NYC, the rest of NYS (excluding NYC), and NYS overall) during the COVID-19 pre-pandemic (2018-2019) and post-pandemic (2020-2021) periods. No significant difference in regional childhood vaccination rates for nine vaccines in NYS existed between the pre-pandemic and post-pandemic periods. Sample sizes: pre-pandemic NYS overall = 1,370; post-pandemic NYS overall = 1,026; pre-pandemic rest of NYS = 507; post-pandemic rest of NYS = 466; pre-pandemic NYC = 863; post-pandemic NYC = 560. =: equal; %: percent; >: greater than; CI: confidence interval; COVID-19: coronavirus disease 2019; NYC: New York City; NYS: New York State; p: probability.

Comparison	Mean difference (%)	95% CI	p-value
NYC vs. NYS overall (pre-pandemic)	0.12	-2.30 to 2.54	>0.9999
NYC vs. NYS overall (post-pandemic)	-1.14	-3.56 to 1.28	0.7393
NYC vs. rest of NYS (pre-pandemic)	0.22	-2.20 to 2.64	0.9998
NYC vs. rest of NYS (post-pandemic)	-2.03	-4.45 to 0.39	0.1533
Rest of NYS vs. NYS overall (pre-pandemic)	-0.11	-2.53 to 2.31	>0.9999
Rest of NYS vs. NYS overall (post-pandemic)	0.88	-1.54 to 3.30	0.8940

**Figure 1:**
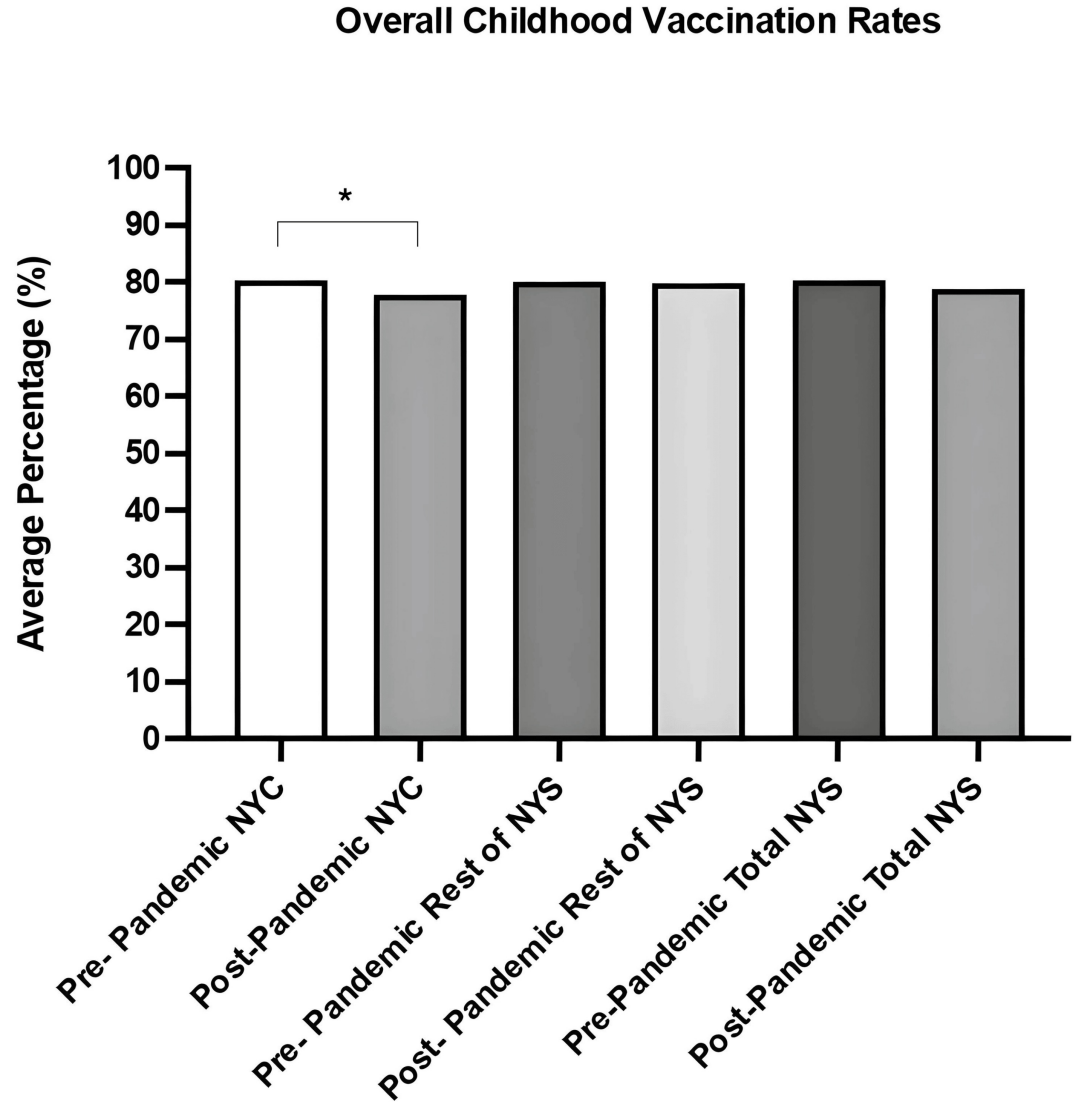
Childhood vaccination rates in NYS for nine vaccines, comparing data before and after the pandemic. This bar graph compares aggregated vaccination rates for the nine recommended vaccines across the three geographic regions before (2018-2019) and after (2020-2021) the onset of the COVID-19 pandemic. The regions include NYC, the rest of NYS (excluding NYC), and NYS (total or overall). Each bar represents the average vaccine coverage for the respective period and location. *The p-value was considered statistically significant if it was less than 0.05. Sample sizes: pre-pandemic NYS overall = 1,370; post-pandemic NYS overall = 1,026; pre-pandemic rest of NYS = 507; post-pandemic rest of NYS = 466; pre-pandemic NYC = 863; post-pandemic NYC = 560. =: equal; %: percent; NYC: New York City; NYS: New York State; p: probability.

See Figure 1 for a visual illustration of the differences between pre-pandemic and post-pandemic periods across NYS, NYC, and the rest of NYS, representing the shifts in overall childhood vaccination uptake for the nine recommended vaccines.

Childhood vaccination rates of the MMR vaccine in NYS pre- and post-pandemic

The statewide MMR vaccination rate experienced a significant decline, decreasing from 93.30% before the pandemic to 89.60% following the pandemic (p = 0.0459). The mean difference was measured at 3.70%. In contrast, the MMR vaccination rate in the rest of NYS showed a slight decrease from 93.25% pre-pandemic to 91.75% post-pandemic, which was not statistically significant (p = 0.5496), resulting in a mean difference of only 1.50%. Notably, the MMR vaccination rate in NYC demonstrated a substantial decline from 93.30% pre-pandemic to 86.70% post-pandemic (p = 0.0039), reflecting a mean difference of 6.60%. Please refer to Table 3 for further details.

**Table 3:** Pre- and post-pandemic childhood MMR vaccination rates in NYS, NYC, and the rest of NYS (excluding NYC). This table shows the average childhood vaccination rates for the MMR vaccine. It reports vaccination rates before (2018-2019) and after (2020-2021) the onset of COVID-19 in NYS overall, NYC, and the rest of NYS (excluding NYC). *The p-value was considered statistically significant if it was less than 0.05. Sample sizes: pre-pandemic NYS overall = 1,370; post-pandemic NYS overall = 1,026; pre-pandemic rest of NYS = 507; post-pandemic rest of NYS = 466; pre-pandemic NYC = 863; post-pandemic NYC = 560. =: equal; %: percent; CI: confidence interval; COVID-19: coronavirus disease 2019; MMR: measles, mumps, and rubella; NYC: New York City; NYS: New York State; p: probability.

Region	Pre-pandemic (%)	Post-pandemic (%)	Mean difference (%)	95% CI	p-value
NYS overall	93.30 (80.59-106.0)	89.60 (81.98-97.22)	3.70	0.08 to 7.32	0.0459*
Rest of NYS	93.25 (76.10-110.4)	91.75 (75.87-107.6)	1.50	-2.12 to 5.12	0.5496
NYC	93.30 (84.41-102.2)	86.70 (82.89-90.51)	6.60	2.98 to 10.22	0.0039*

In examining specific regions, initial comparisons of pre-pandemic MMR vaccination rates indicated no significant differences, with p-values exceeding 0.9999. However, the post-pandemic analysis revealed a remarkable disparity between NYC and the rest of NYS, highlighting a notable decrease in MMR vaccination rates in NYC with a p-value of 0.0128, corresponding to a mean difference of -5.05%. Please access Table 4 for additional information.

**Table 4:** Regional childhood MMR vaccination rates in NYS with pre- and post-pandemic comparisons. This table provides a comparative regional analysis of childhood MMR vaccination rates before (2018-2019) and after (2020-2021) the COVID-19 pandemic. It highlights changes in vaccine uptake and statistically significant disparities, especially between NYC and the rest of NYS (excluding NYC) post-pandemic. *The p-value was considered statistically significant if it was less than 0.05. Sample sizes: pre-pandemic NYS overall = 1,370; post-pandemic NYS overall = 1,026; pre-pandemic rest of NYS = 507; post-pandemic rest of NYS = 466; pre-pandemic NYC = 863; post-pandemic NYC = 560. =: equal; %: percent; >: greater than; CI: confidence interval; COVID-19: coronavirus disease 2019; MMR: measles, mumps, and rubella; NYC: New York City; NYS: New York State; p: probability.

Comparison	Mean difference (%)	95% CI	p-value
NYC vs. NYS overall (pre-pandemic)	0.00	-3.62 to 3.62	>0.9999
NYC vs. NYS overall (post-pandemic)	-2.90	-6.52 to 0.72	0.1103
NYC vs. rest of NYS (pre-pandemic)	0.05	-3.57 to 3.67	>0.9999
NYC vs. rest of NYS (post-pandemic)	-5.05	-8.67 to -1.43	0.0128*
Rest of NYS vs. NYS overall (pre-pandemic)	-0.05	-3.67 to 3.57	>0.9999
Rest of NYS vs. NYS overall (post-pandemic)	2.15	-1.47 to 5.77	0.2662

**Figure 2:**
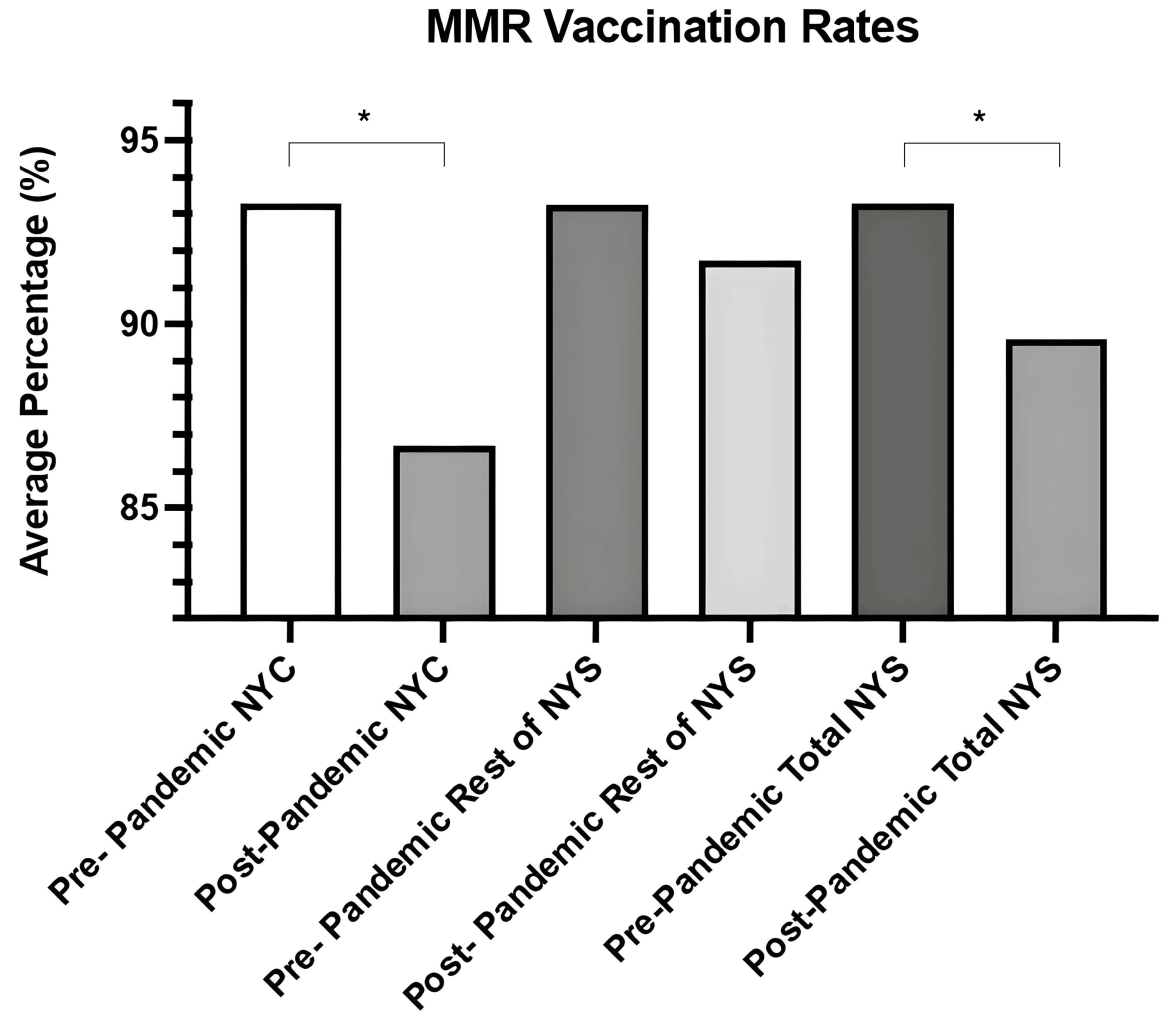
Childhood MMR vaccination rates in NYS for nine vaccines, comparing data before and after the pandemic. This figure visualizes the childhood MMR vaccination rates before (2018-2019) and after (2020-2021) the pandemic in NYS overall (total), NYC, and the rest of NYS (excluding NYC). The graphical representation underscores significant regional disparities and reductions in MMR coverage post-pandemic, particularly in NYC compared to the rest of NYS. *The p-value was considered statistically significant if it was less than 0.05. Sample sizes: pre-pandemic NYS overall = 1,370; post-pandemic NYS overall = 1,026; pre-pandemic rest of NYS = 507; post-pandemic rest of NYS = 466; pre-pandemic NYC = 863; post-pandemic NYC = 560. =: equal; %: percent; MMR: measles, mumps, and rubella; NYC: New York City; NYS: New York State; p: probability.

For visual illustration, refer to Figure 2, which displays pre- and post-pandemic MMR vaccination rates across the same geographic subdivisions. It highlights the significant decline observed, particularly within NYC.

## Discussion

The resurgence of measles in the US is a stark reminder of the critical need to maintain robust childhood vaccination coverage. These outbreaks underscore the urgent need to close immunization gaps and emphasize the need to restore public confidence in the safety and effectiveness of vaccines. This study revealed a concerning decline in overall childhood vaccination rates in NYC, the rest of NYS (excluding NYC), and the overall NYS following the onset of the COVID-19 pandemic, with the only statistically significant outcome being in NYC. In NYC, the average vaccination rates dropped from 80.41% pre-pandemic to 77.82% post-pandemic (p = 0.0288).

Similarly, MMR vaccine coverage declined across all regions, with statistically significant decreases in the NYS and NYC regions. In NYS overall, MMR coverage fell from 93.30% to 89.60% (p = 0.0459), while in NYC, the drop was more pronounced-from 93.30% to 86.70% (p = 0.0039). Furthermore, there was a statistically significant difference in post-pandemic comparison between NYC and the rest of NYS, with a more substantial decline in NYC. The findings indicate that while the pandemic exerted widespread effects, specific regions, most notably NYC, experienced more profound and disruptive declines in immunization rates. This trend warrants serious consideration and concern.

The sharper decrease observed in NYC likely reflects a combination of region-specific factors. The city endured an early and severe COVID-19 wave, resulting in prolonged lockdowns and heightened public concern about in-person healthcare visits and disruption of healthcare services [24]. Additionally, NYC’s reliance on clinics in high-density population centers and public transportation may have created further barriers for families accessing pediatric care [25]. The strain placed on healthcare systems during the pandemic likely compounded these challenges, reducing the availability and prioritization of preventive services, including vaccinations [13]. These factors, combined with potential issues such as vaccine hesitancy and misinformation, could have contributed to the more significant decline in NYC [26].

Although the rest of NYS also saw a reduction in vaccination rates, the decline was more modest. It may be due to differences in healthcare infrastructure, patterns of healthcare utilization, or demographic and socioeconomic variables influencing healthcare access and behavior. These findings suggest that urban areas with high population density and system strain may be particularly vulnerable to drops in routine immunization during public health crises [27].

The public health implications of these declines are significant. Even small reductions in vaccination coverage can compromise herd immunity, increasing the risk of vaccine-preventable diseases such as measles outbreaks. The results reinforce the need for targeted recovery strategies in regions like NYC, where declines were most severe [28]. These strategies, which may include catch-up vaccination initiatives, enhanced public messaging about vaccine safety, and policies that strengthen healthcare delivery systems during emergencies, are of utmost importance [29].

The trends observed in NYS mirror broader national and global patterns. Worldwide, childhood vaccination rates declined during the COVID-19 pandemic due to disruptions in healthcare systems and reduced access to routine services [19]. In the US, the declaration of a national emergency in March 2020 shifted public health priorities, resulting in a drop in vaccine orders through the VFC program [13]. From a population health standpoint, even modest reductions in immunization coverage can facilitate disease resurgence [30].

Multiple factors likely contributed to the decline in vaccination coverage post-pandemic. These include rising vaccine hesitancy fueled by misinformation, erosion of trust in public health authorities, and logistical barriers to accessing care, which require effective remediation strategies [31]. The pandemic also deepened existing disparities, disproportionately affecting low-income and minority populations. Widespread misinformation, particularly via social media, further complicated public perceptions of routine childhood immunizations during this period [32].

The decline in childhood vaccination rates in NYS, particularly the significant decreases observed in NYC and in MMR coverage statewide, reflects the broader impacts of the COVID-19 pandemic on preventive healthcare. These findings highlight the need for regionally tailored public health strategies to restore [33].

Clinical implications

The observed decline in childhood vaccination rates after the pandemic emphasizes the urgent need to target vaccination efforts in pediatric populations. It is vital in areas with significant declines, such as NYC. Healthcare providers should proactively identify under-immunized or non-immunized children during visits and use that time to educate families on the importance of vaccinations.

Another critical implication from this study is reinforcing vaccination confidence in families. Pediatricians and other primary care providers are essential in addressing vaccine hesitancy. The disruptions and false narratives about vaccinations caused by the pandemic may have deepened mistrust or confusion around routine immunizations. Clear and evidence-based communication by clinicians is essential to rebuild confidence and improve uptake.

Lastly, another clinical implication is the importance of surveillance and documentation of immunization status. By tracking the number of immunized people, healthcare providers and public health organizations can monitor trends and identify areas that need more support.

Study limitations

We encountered a few challenges during this research. Firstly, the study relied exclusively on publicly available data from the CDC, which were provided solely as percentages without access to individual-level data. This restriction constrained our ability to calculate measures of variability, such as standard deviation, and to adjust for potential confounders, including socioeconomic status, healthcare access, or regional differences in infrastructure.

Secondly, the ecological nature of the study, based on aggregated population-level data, restricts the ability to conclude individual behaviors or causality. Important factors influencing vaccine uptake-such as parental hesitancy, provider recommendations, and access to care-could not be assessed directly.

Lastly, using only two broad timeframes, pre-pandemic (2018-2019) and post-pandemic (2020-2021), may risk oversimplifying the intricate timeline of changes. Vaccination rates were likely affected by multiple waves of the pandemic, shifting public health guidance, school reopening strategies, and variations in vaccine misinformation. Aggregating data into two distinct periods may have concealed more nuanced, year-to-year trends in vaccine coverage.

Future indications

This study elucidates trends in childhood vaccination rates across NYS, with a particularly significant decline in NYC following the COVID-19 pandemic. Key immunizations, including the MMR vaccine, have exhibited marked decreases, heightening the risk of future outbreaks of vaccine-preventable diseases. These trends indicate broader systemic challenges, encompassing disrupted access to healthcare, increased vaccine hesitancy, and the proliferation of misinformation during the pandemic. Future investigations must identify the root causes of these declines, especially in NYC, to better understand specific healthcare requirements and guide focused public health strategies.

Addressing these declines necessitates a multifaceted strategy. Public health initiatives should focus on restoring routine immunization coverage through targeted outreach in under-vaccinated communities, enhancing education to rebuild vaccine trust, and incorporating vaccination services into educational institutions and community clinics. Healthcare providers can contribute significantly by implementing proactive vaccine tracking and reminder systems, while policymakers may need to reinforce school-entry vaccination mandates and support catch-up vaccination initiatives. These combined efforts are vital for preventing the resurgence of preventable diseases and ensuring the overall health of NYS’s pediatric population.

## Conclusions

The findings of this study suggest that the COVID-19 pandemic may have contributed to declining childhood vaccination rates in NYS, with more pronounced reductions observed in NYC, particularly concerning MMR coverage. While overall vaccination rates showed modest decreases, urban areas like NYC have faced more significant challenges, possibly related to healthcare access barriers and misinformation. These findings raise important questions about the factors influencing vaccination rates during the pandemic and the potential need for targeted interventions. Addressing these issues could be essential for restoring vaccination coverage, improving vaccine confidence, and implementing effective catch-up programs. If left unaddressed, the observed trends could risk herd immunity and potentially lead to outbreaks of vaccine-preventable diseases such as measles.
